# Positron emission tomography and magnetic resonance imaging of the brain in experimental human malaria, a prospective cohort study

**DOI:** 10.1038/s41598-022-09748-y

**Published:** 2022-04-05

**Authors:** John Woodford, Ashley Gillman, Peter Jenvey, Jennie Roberts, Stephen Woolley, Bridget E. Barber, Melissa Fernandez, Stephen Rose, Paul Thomas, Nicholas M. Anstey, James S. McCarthy

**Affiliations:** 1 Clinical Tropical Medicine Laboratory, QIMR-Berghofer Medical Research Institute, Brisbane, QLD 4029 Australia; 2 University of Queensland, Brisbane, QLD 4006 Australia; 3 Australian e-Health Research Centre, Commonwealth Scientific and Industrial Research Organisation, Brisbane, QLD 4006 Australia; 4 Department of Radiology, Royal Brisbane and Women’s Hospital, Brisbane, QLD 4029 Australia; 5 Herston Imaging Research Facility, Brisbane, QLD 4006 Australia; 6 Global and Tropical Health Division, Menzies School of Health Research and Charles Darwin University, Darwin, NT 0811 Australia

**Keywords:** Experimental models of disease, Parasite biology, Parasite host response

## Abstract

Cerebral malaria is the most serious manifestation of severe falciparum malaria. Sequestration of infected red blood cells and microvascular dysfunction are key contributing processes. Whether these processes occur in early stage disease prior to clinical manifestations is unknown. To help localize and understand these processes during the early stages of infection, we performed 18-F fluorodeoxyglucose positron emission tomography/magnetic resonance imaging in volunteers with *Plasmodium falciparum* induced blood stage malaria (IBSM) infection, and compared results to individuals with *P. vivax* infection, in whom coma is rare*.* Seven healthy, malaria-naïve participants underwent imaging at baseline, and at early symptom onset a median 9 days following inoculation (n = 4 *P. falciparum*, n = 3 *P. vivax*). Participants with *P. falciparum* infection demonstrated marked lability in radiotracer uptake across all regions of the brain, exceeding expected normal variation (within subject coefficient of variation (wCV): 14.4%) compared to the relatively stable uptake in participants with *P. vivax* infection (wCV: 3.5%). No consistent imaging changes suggestive of microvascular dysfunction were observed in either group. Neuroimaging in early IBSM studies is safe and technically feasible, with preliminary results suggesting that differences in brain tropism between *P. falciparum* and *P. vivax* may occur very early in infection.

## Introduction

Severe malaria causes significant global morbidity and mortality^1^. In *Plasmodium falciparum* infection*,* organ-specific sequestration of infected red blood cells and microvascular dysfunction are believed to be key mechanisms underlying severe disease, including the critical complication of cerebral malaria^2,3^. *P. falciparum* virulence and tissue tropism of infected red blood cells is believed to be mediated by expression of specific adhesion molecules on the surface of erythrocytes that mediate sequestration in tissues with the cognate ligand. Adhesion molecule/ligand combinations associated with severe organ-specific disease have been described post-mortem^4^, and more recently in life, correlated with clinical cerebral malaria on imaging^5^. In contrast, *P. vivax* adheres significantly less frequently to microvascular endothelial cells^6^, and coma is rare^7^, although generalized endothelial activation and microvascular dysfunction related to systemic inflammation are common^8,9^. For both species, headache is frequently an early symptom of clinical infection, but its pathophysiology is poorly understood. Understanding the upstream processes contributing to the cerebral complications of malaria may be useful to help design interventions that may improve clinical outcomes.

Neuroimaging studies in uncomplicated and cerebral malaria have led to the identification of a spectrum of changes ranging from localized restricted diffusion in adults hospitalized with uncomplicated malaria^10,11^, to significantly increased cerebral volume that may predict a fatal outcome^12–14^. Non-human primate studies of cerebral malaria have demonstrated decreased glucose uptake in the anterior circulation of the brain in symptomatic and neurologically intact macaques prior to any parenchymal damage, and not strictly collocated with sequestered parasites at necropsy^15^. No studies have been performed in earlier stage malaria infection. We have previously used 18-F fluorodeoxyglucose positron emission tomography and magnetic resonance imaging (FDG PET/MRI) to demonstrate differences between *P. falciparum* and *P. vivax* in splenic tropism, volume and glucose metabolism in early experimental malaria^16^, and now present the neuroimaging findings from this study cohort.

We performed PET/MRI prior to infection and at symptom onset in participants with experimentally induced blood stage malaria (IBSM). In this study, we sought to investigate if changes in overall glucose metabolism in the brain or in the cerebral circulation were present in early stage infection. We compared findings in participants infected with *P. falciparum* to those infected with *P. vivax*.

## Results

### Study participants and IBSM studies

A total of 8 healthy, malaria-naïve participants were recruited. All participants underwent baseline imaging within a 7-day interval before inoculation. One participant was enrolled in the imaging study and underwent baseline imaging but was excluded from the IBSM study prior to inoculation due to hypokalemia. This participant did not undergo post-inoculation imaging and was excluded from the analysis. Thus, seven participants underwent post-inoculation imaging. The study population is described in Table 1. Included participants had a median age of 20 years (range 19–23), and 6/7 were male. Three were inoculated with *P. vivax,* and four with *P. falciparum*. All participants developed a measurable parasitemia with expected growth kinetics (Supplementary Fig. S1)^17^, and reported symptoms of early malaria infection prior to treatment. Median parasitemia at post-inoculation imaging was 22,326 (range 998–99,134 parasites/mL) in the *P. falciparum* group and 6042 (range 5818–29,097) parasites/mL in the *P. vivax* group. Although the day of treatment varied between studies, both groups underwent imaging a median 9 days following inoculation. No adverse events related to imaging were recorded and in safety reporting no incidental findings required follow up.

**Table 1:** Study population information.

Participant	Age (years)	Sex	BMI	Trial	Challenge agent	Dose (viable p/mL)	Day of post inoculation imaging	Parasitemia at imaging (p/mL)
1	20	F	29.5	Collins et al. 2020^18^	Pv	564	9	29,097
2	23	M	22.9	Collins et al. 2020^18^	Pv	564	9	5818
3	20	M	22.0	Collins et al. 2020^18^	Pv	564	9	6042
4	19	M	28.5	Gaur et al. 2020^19^	Pf	2800	7	1427
5	22	M	22.0	Gaur et al. 2020^19^	Pf	2800	7	998
6	19	M	19.7	Woolley et al. 2021^20^	Pf	2800	11	43,224
7	19	M	24.2	Woolley et al. 2021^20^	Pf	2800	10	99,134

Parasitemia at imaging: estimated by interpolating the linear rate of change between the closest log-transformed parasitemia measurements.

*BMI* body mass index, *Pv* *P. vivax,* *Pf* *P. falciparum.*

### Clinical review

Regular physical examinations, and clinician-elicited and self-reported symptoms were recorded for all participants. Safety laboratory results have been reported previously^16^. All participants reported some symptoms consistent with early malaria infection, and the majority reported headache. All participants in the *P. vivax* group reported mild headache during the course of the study. Participant 1 reported an intermittent episode, starting the day prior to post-inoculation imaging, and resolving two days following imaging. Participant 2 and 3 reported headache starting on the day of post-inoculation imaging, and resolving four days following, and on the same day of imaging respectively. No other neurological symptoms or abnormal neurological examinations were recorded.

In the *P. falciparum* group, most participants reported mild headache prior to treatment. Participant 5 reported a headache on the day prior to imaging. Participants 6 and 7 reported intermittent episodes, commencing the day of, and the day prior to post-inoculation imaging, and resolving two days and one day following imaging respectively. Participant 4 reported an episode of mild dizziness that resolved 6 days prior to post-inoculation imaging. No other neurological symptoms or abnormal neurological examinations were recorded.

### Imaging

All participants underwent matched baseline and post-inoculation neuroimaging protocols. With the exception of Participant 4, all participants had a radiotracer uptake time of approximately 120 min before commencement of neurological PET (Supplementary Table S1). The mean SUV was estimated for all regions of interest, and ADC was estimated for the splenium of the corpus callosum for all participants, for both imaging episodes.

**Figure 1:**
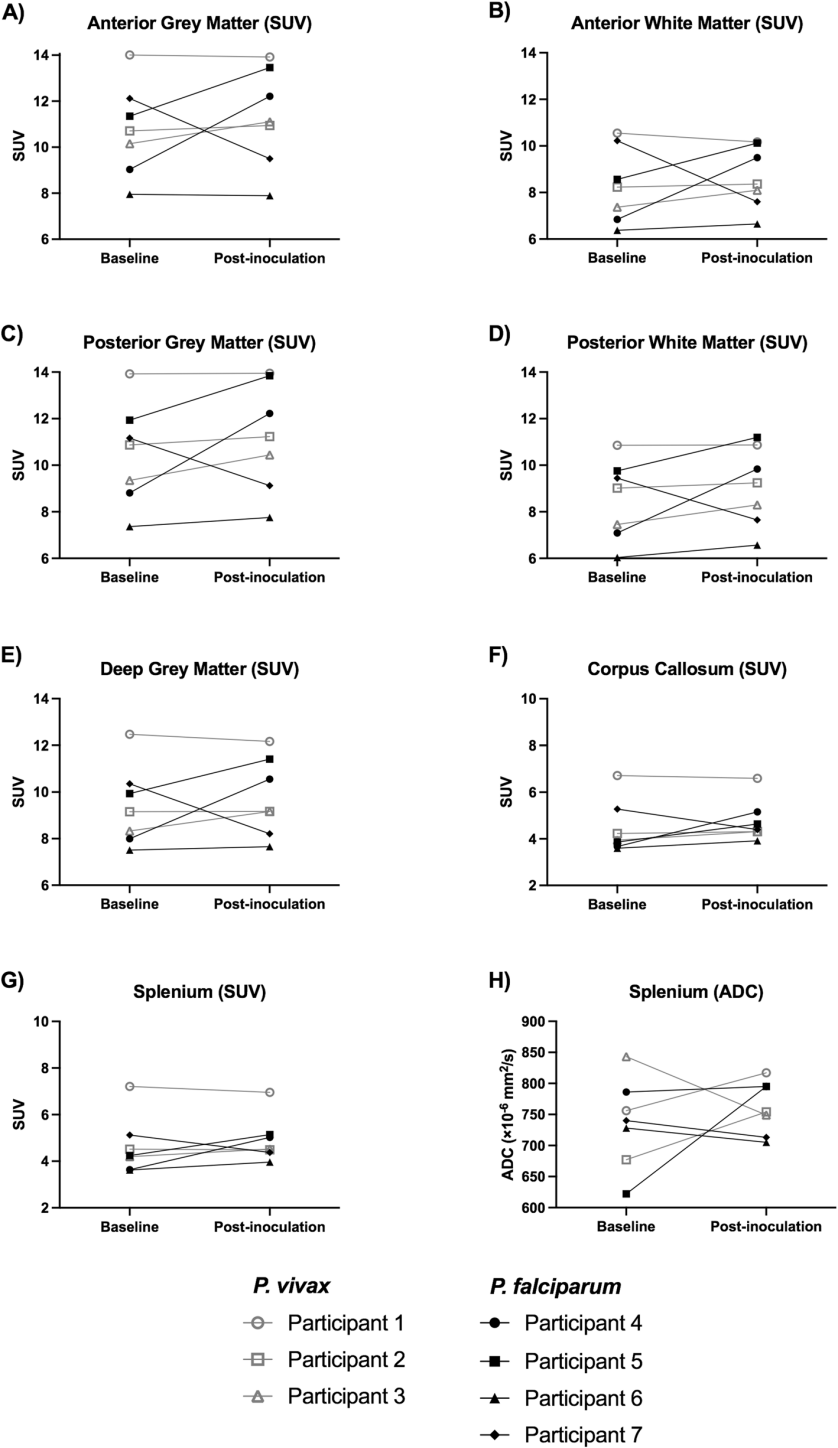
Imaging indices at baseline and post-inoculation. (**A**) Anterior grey matter SUV, (**B**) Anterior white matter SUV, (**C**) Posterior grey matter SUV, (**D**) Posterior white matter SUV, (**E**) Deep grey matter SUV, (**F**) Corpus callosum SUV, (**G**) Splenium SUV, (**H**) Splenium ADC, SUV: mean standardized uptake value, ADC: apparent diffusion coefficient. *P. vivax* participants represented by grey lines with unfilled markers, *P. falciparum* participants represented by black lines with filled markers.

In the *P. vivax* group, the within subject coefficient of variation (wCV) for radiotracer uptake across all brain segments was 3.5%, within the defined 10% range of normal inter-scan variability (Table 2). Change in radiotracer on post-inoculation imaging was consistent across all subregions of the brain. Only Participant 3 experienced a change in radiotracer uptake greater than 10% in any subregion (Fig. 1, Supplementary Table S2). The mean SUV change from baseline was + 3.6% in the anterior grey matter (CI: −9.1% to + 16.4%, *p* = 0.354), + 2.6% in the anterior white matter (CI: −14.2% to 19.4%, *p* = 0.667), + 5.1% in the posterior grey matter (CI: −9.6% to + 19.7%, *p* = 0.257), + 4.6% in the posterior white matter (CI: −9.9% to + 19.2%, *p* = 0.282), + 2.6% in the deep grey matter (CI: −13.8% to + 19.0%, *p* = 0.644), and + 3.1% in the corpus callosum (CI: −10.8% to + 17.1%, *p* = 0.516). In the splenium of the corpus callosum the mean SUV change was + 1.2% (CI: −13.3% to + 15.9%, *p* = 0.929) and the mean ADC (representing the free movement of water) change was + 2.8% (CI: −27.5% to + 33.0%, *p* = 0.813). There were no changes suggestive of microvascular dysfunction on qualitative review.

**Table 2:** Within subject coefficient of variation (wCV) for radiotracer uptake in each subregion of the brain and overall brain in *P.vivax* and *P. falciparum* groups.

	Within subject coefficient of variation (wCV, %)	
Subregion	*P. vivax*	*P. falciparum*
Anterior grey matter SUV	3.5	16.6
Anterior white matter SUV	4.6	18.6
Posterior grey matter SUV	3.9	15.5
Posterior white matter SUV	3.9	15.6
Deep grey matter	4.5	15.2
Corpus callosum SUV	3.8	15.4
Splenium SUV	4.1	14.2
Overall brain SUV	3.5	14.4

*SUV* standardized uptake value.

**Figure 2:**
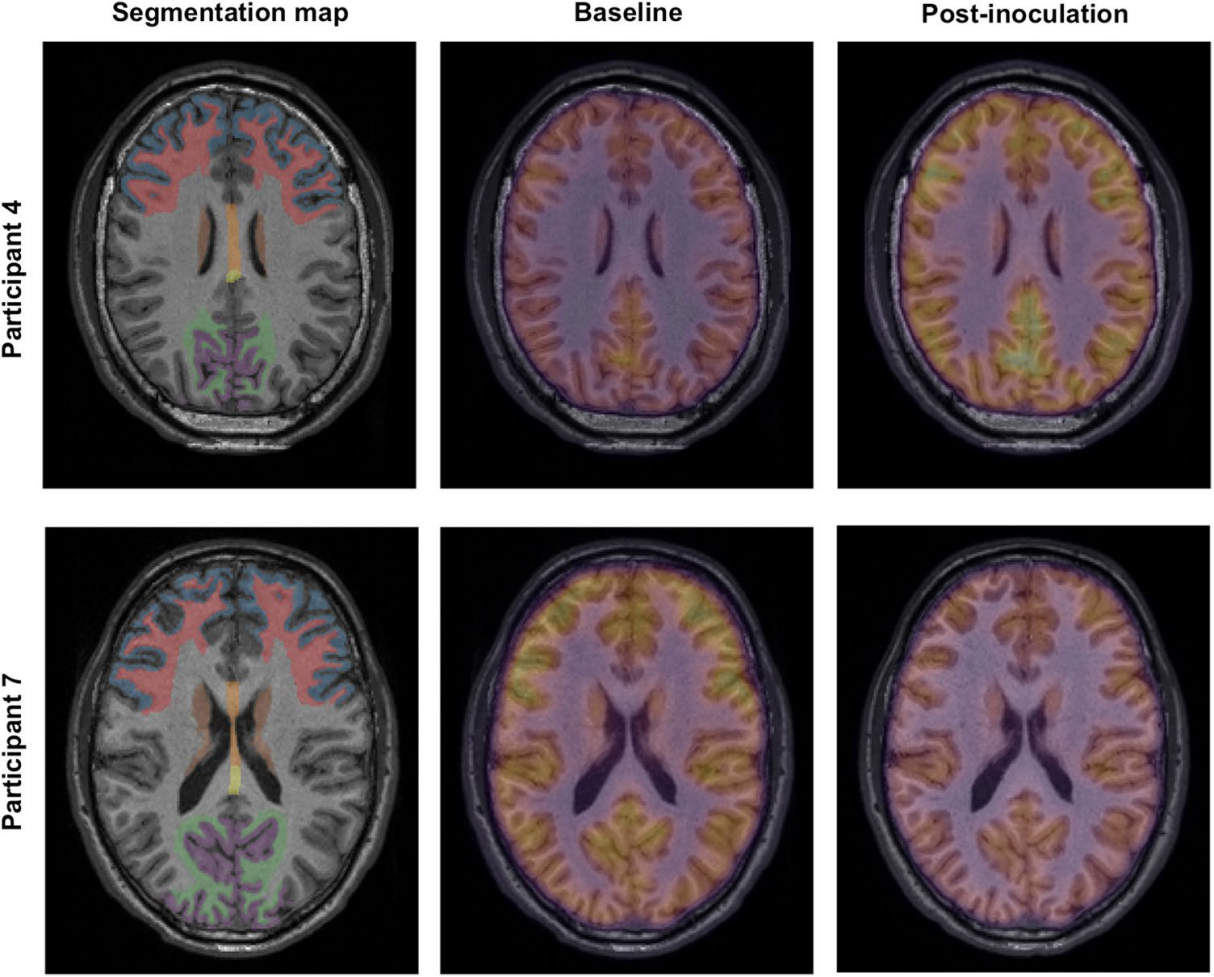
Example posterior subregion segmentations of T1-weighted MRI sequence (left panels), whole brain radiotracer uptake at baseline (middle panels) and post-inoculation (right panels) in Participant 4 (top row) and Participant 7 (bottom row). Segmentation map: blue—anterior grey matter; red—anterior white matter; brown—deep grey matter; yellow/orange—corpus callosum (yellow indicates splenium); purple—posterior grey matter; green—posterior white matter. Participant 4: Increased radiotracer uptake is evident globally on post-inoculation imaging. Participant 7: Decreased radiotracer uptake is evident globally on post-inoculation imaging.

In the *P. falciparum* group, the wCV for radiotracer uptake across brain segments was 14.4% (Table 2), exceeding the expected inter-scan variability in the presence of no intervention. For each participant these changes in radiotracer uptake were consistent across different subregions of the brain. Participants 4 and 5 demonstrated consistently increased SUVs from baseline, Participant 6 demonstrated stable SUVs, and Participant 7 demonstrated consistently decreased SUVs (Fig. 1, Supplementary Table S2). Some of these changes were apparent on qualitative review (Fig. 2). The mean SUV change was + 7.9% in the anterior grey matter (CI: −31.2% to + 46.9%, *p* = 0.645), + 9.0% in the anterior white matter (CI: −34.1% to + 52.0%, *p* = 0.708), + 10.4% in the posterior grey matter (CI: −27.3% to + 48.2%, *p* = 0.488), + 10.9% in the posterior white matter (CI: −26.9% to + 48.6%, *p* = 0.499), + 7.0% in the deep grey matter (CI: −28.4% to + 42.3%, *p* = 0.652), and + 13.2% in the corpus callosum (CI: −24.7% to + 51.1%, *p* = 0.455). In the splenium of the corpus callosum the mean SUV change was + 13.5% (CI: −21.6% to + 48.6%, *p* = 0.379) and the mean ADC change was + 5.5% (CI: −18.3% to + 29.4%, *p* = 0.536); however, Participant 5 experienced an increase in ADC of 27.8%, representing a relative increase in the free movement of water. There were no changes suggestive of microvascular dysfunction on qualitative review and no apparent relationship between parasitemia and radiological findings. The mean percentage change from baseline for reported quantitative imaging metrics was not statistically different between *P. falciparum* and *P. vivax* groups (Supplementary Table S3).

## Discussion

Simultaneous FDG PET/MRI of the brain in early experimental human malaria infection demonstrated labile glucose uptake in 3 of 4 participants with *P. falciparum* infection and limited variability post-inoculation in participants with *P. vivax* infection. Although changes were not statistically different between baseline and post-inoculation imaging in either group, radiotracer uptake exceeded expected inter-scan variability in the *P. falciparum* group but was within normal limits in the *P vivax* group. This may be related to the known cerebral tropism of *P. falciparum* compared to *P. vivax,* and suggests that differences in brain tropism between *P. falciparum* and *P. vivax* may occur very early in infection*.* In both groups, there was no apparent relationship between radiotracer uptake and neurological symptoms, specifically headache, and no consistent radiographic evidence of cerebral microvascular dysfunction. This is the first examination of cerebral glucose metabolism in early experimental human malaria infection.

While changes in radiotracer uptake are challenging to attribute completely to experimental malaria infection, variability between baseline and post-inoculation imaging was controlled as much as reasonably possible in this study. All participants were screened for impaired fasting glucose at enrollment, had similar dietary and fasting conditions between scans, and underwent the same imaging protocol under the same conditions for paired baseline and post-inoculation imaging. Therefore intra-individual variation is most likely related to experimental malaria. Nonetheless, we are unable to completely exclude other factors that may influence brain glucose consumption such as uncontrolled use of caffeine or alcohol^21^.

As FDG is a non-specific indicator of intracellular glucose accumulation, changes in uptake following inoculation may be related to parasite or host activity. Non-human primate PET studies have identified intense FDG uptake associated with accumulation of parasites in the spleen^22^, and we have recently described enhanced splenic uptake in the same population of participants following both experimental *P. falciparum* and *P. vivax* infection. In the latter study splenic uptake was significantly increased in *P. vivax* compared to *P. falciparum* infection*,* suggesting splenic tropism with this species^16,23^. In the current study, interval change in radiotracer uptake was largely uniform across all brain subregions, irrespective of blood supply or white–grey matter differentiation for all participants. This is in contrast to the pattern of parasite sequestration described at autopsy following severe *P. falciparum* disease, where parasite distribution is heterogeneous with a predilection for the grey matter of the brain^4,24^. It is not clear the extent to which global alterations in brain radiotracer uptake are related directly to parasite glucose utilization in this study.

Globally increased radiotracer uptake may be related to generalized host cell activation throughout the brain or alterations in blood flow and neuronal metabolism, both of which may be stimulated by local or circulating parasite and host factors. Cerebral endothelial activation or activation of other host cells by a small number of parasites or inflammatory mediators may contribute to the generalized increase in radiotracer uptake observed in early infection. We and others have previously demonstrated systemic endothelial cell activation in human experimental malaria^9,25^, and direct contact with infected red blood cells stimulates microglial proliferation prior to the development of cerebral malaria in mice^26^. In the healthy state, the majority of glucose consumption in the brain reflects normal synaptic activity. Changes in overall cerebral blood supply, blood glucose, or brain activity may result in alterations to radiotracer uptake^21^, and uncomplicated malaria has been associated with increased glucose production^27^. Notably, there was no MRI evidence of microvascular dysfunction or restricted diffusion in either group in this study. Similar imaging protocols have previously demonstrated restricted diffusion in the splenium of the corpus callosum in a moderate proportion of patients presenting with uncomplicated *P. falciparum* infection^10,11^; however, our observations suggest that these changes are not present or not detectable in the earliest stages of infection.

Of the three participants that experienced a change in radiotracer uptake following *P. falciparum* inoculation, two experienced an increase (Participant 4 and 5) and one a decrease (Participant 7). Increased uptake may be attributed to host cell activation or increased blood flow, while decreased uptake may be related to reduced blood flow, hyperglycemia, or an otherwise generalized reduction in metabolism. Decreased regional glucose uptake has been reported in primate models of cerebral malaria^15^, suggesting parasite may affect one or some of these mechanisms. Interestingly, in our study uptake decreased in the Participant 7, who had the highest circulating *P. falciparum* parasitemia, and increased in the participants with lowest (Participants 4 and 5). Although there were no consistent changes in splenial diffusion in either inoculum group, Participant 4 experienced an increase in ADC suggestive of vasogenic edema, which may reflect a change to cerebral blood flow in this individual. We have previously shown abdominal radiotracer uptake changes following experimental malaria infection restricted to the spleen in this cohort^16^. As a result, changes observed in the brain are an organ-specific rather than global phenomenon.

Although the study population is small, our findings demonstrate that PET/MRI is feasible in early experimental human malaria infection and the pattern of FDG uptake appears different in *P. falciparum* compared to *P. vivax* infection. Nonetheless, there are several limitations. Firstly, the small study population size means we were not able to demonstrate a statistical difference between quantitative imaging metrics, only that changes in the *P. falciparum* group exceed expected inter-scan variability. Although paired imaging has been rigorously standardized, there may be residual concerns about inter-scan variability, the lower limits of detection, and sensitivity of the imaging protocol. Secondly, FDG is non-specific and not able to differentiate between host and parasite activity. Advances in radiochemistry may allow for the future development of parasite-specific radiotracers. Thirdly, we were not able to evaluate the influence of cerebral blood flow on radiotracer uptake. The relative contribution of cerebral blood flow on radiotracer uptake following inoculation could potentially be examined using emerging imaging sequences such as resting-state blood oxygen level dependent functional MRI with simultaneous PET^28^, although the applicability of such a protocol has not previously been evaluated. Finally, as a study of early experimental human infection, the dynamic range of parasitemia and disease severity is greatly restricted compared to clinical disease. As a result, it is difficult to infer any relationship between parasitemia and uptake in our small cohort.

There is a paucity of literature examining the organ-specific pathophysiology of early human malaria infection. Understanding upstream events may help us understand the processes contributing to later disease, which may be particularly important for the critical complication of cerebral malaria. We have demonstrated that PET/MRI is feasible in early experimental malaria infection, and that brain glucose metabolism may be more affected by *P. falciparum* infection compared to *P. vivax* infection, but these changes are not uniform between individuals. This is in keeping with known species-specific tissue tropism and later stage disease complications, and consolidates the notion that these imaging techniques may offer useful insights into disease pathophysiology.

## Methods

### Study participants and procedures

This was a prospective, single-center exploratory study performed between 2016 and 2019 at the Herston Imaging Research Facility, Brisbane, Australia. Baseline imaging followed by experimental malaria inoculation and post-inoculation imaging was performed as previously described^16^. In brief, baseline PET/MRI was performed in the 7 days prior to malaria inoculation on healthy malaria-naïve adults aged 18–55 years participating in IBSM studies. Following inoculation with approximately 2800 viable *P. falciparum* or approximately 564 viable *P. vivax* infected red blood cells, peripheral blood parasitemia was monitored at least daily by qPCR^29^. Post-inoculation imaging was performed near the peak of parasitemia, in the 24 h prior to administration of antimalarial treatment. In addition to standard IBSM study inclusion criteria, participants were required to have a normal fasting glucose and no contraindications to MRI. Due to protocol-specified differences in the contributing studies, the interval between inoculation and post-inoculation imaging varied between studies. Standard safety assessments including regular physical examinations, and clinician-elicited and self-reported symptoms were performed at specified times during the IBSM studies. A total of eight participants were planned for enrollment, based on the availability of funding, IBSM study cohorts, and imaging facilities. The imaging study, and all IBSM studies contributing participants were performed in accordance with the Declaration of Helsinki.

### Imaging procedures

Baseline and post-inoculation FDG PET/MRI were performed on all participants using the 3 Tesla Magnetom Biograph mMR (Siemens, Erlangen, Germany) at the Herston Imaging Research Facility. Detailed methods and imaging protocols are outlined in the Supplementary Methods. All participants completed an institutional MRI safety checklist and the female participant underwent qualitative human chorionic gonadotropin testing to exclude pregnancy prior to imaging. Participants were required to have a normal fasting glucose at enrollment, abstain from strenuous exercise and follow a low carbohydrate diet in the 24-h prior to imaging with a 6-h fasting period immediately prior to help standardize FDG uptake^30,31^. Diet and activity information sheets were provided to assist participant adherence. Participants were inoculated with a standard dosage of approximately 4.0 MBq/kg of the radiotracer FDG prior to PET imaging. No MRI contrast medium was administered.

Imaging was reported qualitatively and with respect to pre-specified quantitative metrics, and was reviewed separately for safety and incidental findings. Post-inoculation imaging was compared to baseline imaging. Reporting nuclear medicine and radiology specialists were not aware of the inoculum species. Mean standardized uptake values (SUVs) were estimated for brain segmentations representing anterior, posterior, and deep regions of interest (anterior grey and white matter, posterior grey and white matter, deep grey matter, and corpus callosum, including a dedicated splenium assessment). SUV is the most commonly used measurement of radiotracer activity, and represents the ratio of tissue radioactivity related to FDG at a given time and the injected dose of radioactivity per kilogram of participant weight. The apparent diffusion co-efficient (ADC) was estimated for a manually defined region of interest in the corpus callosum representing the splenium, as restricted diffusion has been reported in this region in cases of uncomplicated *P. falciparum* infection^10,11^. ADC represents the relative mobility of water in the tissue, and changes may reflect pathology including relative ischemia^13^.

### Statistical analysis

Imaging results are presented for the *P. vivax* and *P. falciparum* groups. Firstly, to determine if inter-scan variability was outside the expected background variability for PET imaging, the within subject coefficient of variation (wCV) was calculated for each region of interest, and for the brain overall, for each inoculum group as previously described^32^. A wCV < / = 10% represents expected normal inter-scan variability in the absence of an intervention^32–34^. In this study, if experimental human malaria affects imaging parameters, wCV should exceed this normal expected variability. Secondly, assuming that experimental human malaria may affect imaging parameters, the mean (95% confidence interval (CI)) percentage change from baseline imaging for each region of interest was calculated, for each inoculum group. Parasitemia at the time of post-inoculation imaging was estimated by interpolating the linear rate of change between the nearest log-transformed study parasitemia measurements. In an exploratory analysis, baseline and post-inoculation imaging metrics for each group were compared using paired, two-tailed t-tests. Differences between the mean percentage change from baseline for *P. vivax* and *P. falciparum* groups were compared using unpaired t-tests. A *p* value < 0.05 was considered significant. No adjustments were made for multiple comparisons.

### Study approval

Participants were recruited from three IBSM studies: two *P. falciparum:* NCT02867059^19^ and ACTRN12619001085167^20^, and one *P. vivax:* ACTRN12616000174482^18^. These studies were approved by the QIMR-Berghofer Medical Research Institute Ethics committee. The exploratory imaging study was approved by the QIMR-Berghofer Medical Research Institute, and Queensland Health Metro North Ethics committees. All participants provided written informed consent for the IBSM studies and the exploratory imaging study.

### Data availability

The datasets generated during and/or analysed during the current study may be available from the corresponding author on reasonable request and with appropriate data sharing agreements in place.

## Supplementary Information

Supplementary Information.
