# Supplementary material for: Positron emission tomography and magnetic resonance imaging of the brain in experimental human malaria, a prospective cohort study
Source: Sci Rep. 2022 Apr 5;12:5696. doi: 10.1038/s41598-022-09748-y (PMC8983718; doi:10.1038/s41598-022-09748-y)
Supplement: Supplementary file 1 — Supplementary Information. [file 41598_2022_9748_MOESM1_ESM.docx]

**TITLE**

Positron emission tomography and magnetic resonance imaging of the brain in experimental human malaria, a prospective cohort study

**AUTHORS**

John Woodford^1,2^ E: [john.woodford@uqconnect.edu.au](mailto:john.woodford@uqconnect.edu.au)

Ashley Gillman^3^ E : [ashley.gillman@csiro.au](mailto:ashley.gillman@csiro.au)

Peter Jenvey^4^ E : [peter.jenvey@health.qld.gov.au](mailto:peter.jenvey@health.qld.gov.au)

Jennie Roberts^4^ E: [jennie.roberts@health.qld.gov.au](mailto:jennie.roberts@health.qld.gov.au)

Stephen Woolley^1^ E: [stephen.woolley@qimrberghofer.edu.au](mailto:stephen.woolley@qimrberghofer.edu.au)

Bridget E Barber^1^ E: [bridget.barber@qimrberghofer.edu.au](mailto:bridget.barber@qimrberghofer.edu.au)

Melissa Fernandez^1^ E: [melissa.fernandez@qimrberghofer.edu.au](mailto:melissa.fernandez@qimrberghofer.edu.au)

Stephen Rose^3^ E: [stephen.rose@csiro.au](mailto:stephen.rose@csiro.au)

Paul Thomas^5^ E : [paul.thomas@health.qld.gov.au](mailto:paul.thomas@health.qld.gov.au)

Nicholas M Anstey^6^ E: [nicholas.anstey@menzies.edu.au](mailto:nicholas.anstey@menzies.edu.au)

James S McCarthy^1,2^ E: [j.mccarthy@uq.edu.au](mailto:j.mccarthy@uq.edu.au)

^1^ Clinical Tropical Medicine Laboratory, QIMR-Berghofer Medical Research Institute, Brisbane, Queensland 4029, Australia

^2^ University of Queensland, Brisbane, Queensland 4006, Australia

^3^ Australian e-Health Research Centre, Commonwealth Scientific and Industrial Research Organisation, Brisbane, Queensland 4006, Australia

^4^ Department of Radiology, Royal Brisbane and Women’s Hospital, Brisbane, Queensland 4029, Australia

^5^ Herston Imaging Research Facility, Brisbane, Queensland 4006, Australia

^6^ Global and Tropical Health Division, Menzies School of Health Research and Charles Darwin University, Darwin, Northern Territory 0811, Australia

**CORRESPONDENCE:** E: [john.woodford@uqconnect.edu.au](mailto:john.woodford@uqconnect.edu.au)

**Supplementary Methods**

**Magnetic Resonance Imaging**

MRI sequences included T1 gradient echo (Magnetization Prepared - RApid Gradient Echo - MPRAGE), T2 turbo spin echo (TSE), T2 3D fast spin echo (Sampling Perfection with Application optimized Contrasts using different flip angle Evolution - SPACE), ultra-short echo time (UTE) for PET attenuation correction and diffusion weighted imaging (DWI) with calculation of maps of the apparent diffusion coefficient (ADC). No contrast medium was administered.

| **Brain MRI sequences** |
| --- |
| PET Acquisition  Dixon PET attenuation scan (unused in presented analysis)  Ultrashort echo time PET attenuation scan  T2 TSE  Diffusion imaging (B0, 64x B1000, 64x B3000)  T1 Magnetization Prepared - RApid Gradient Echo (MPRAGE)  T2 Sampling Perfection with Application optimized Contrasts using different flip angle Evolution (SPACE) |

**Positron Emission Tomography**

Radiotracer was manufactured on site at the Royal Brisbane and Women’s Hospital Department of Nuclear Medicine as per Good Manufacturing Practice. Participants were required to abstain from strenuous exercise and follow a low carbohydrate diet in the 24-hours prior to imaging with a 6-hour fasting period immediately prior to help standardize FDG uptake [1, 2]. Diet and activity information sheets were provided to assist participant adherence. Following administration of radiotracer, participants underwent static neurological PET following a consistent uptake time for each participant to help standardize FDG uptake [3]. Neurological PET images were reconstructed to 2.1 x 2.1 x 2.0 mm (LRxAPxSI) in 20 dynamic time frames (8 x 15s, 2 x 30s, 2 x 60s, 8 x 300s) using ordered-subsets expectation maximization with corrections for randoms, scatter, normalization, attenuation, decay, and partial volume (HD-PET, Siemens, USA). Gamma-photon MR attenuation correction was performed using ultra short echo time sequences (UTE) [4].

**Imaging reporting procedure**

Nuclear medicine and radiology specialists reporting all imaging were not aware of the inoculum species. Due to electronic date stamping of imaging data, it was not possible to blind the readers of the baseline and post-inoculation images. An external radiologist and a nuclear medicine physician reviewed all MRI and PET imaging for incidental findings, and for safety assessment as per local guidelines. Post-inoculation imaging was reviewed with respect to baseline imaging. A dedicated neuro-radiologist (JR) assessed brain MRI sequences specifically for changes including vascular integrity. A circular region of interest (ROI) representing the splenium was manually placed on ADC maps of the corpus callosum using Inteleviewer (Intelerad, Australia) to measure quantitative change in ADC from baseline to detect altered diffusion, given that restricted diffusion in the splenium has been reported in uncomplicated *P. falciparum* infection [5, 6]. Brain segmentation was performed automatically using CapAIBL (AG) [7] by co-registering the PET reconstruction with the MPRAGE image from the same session, and registering the pair to the FreeSurfer *wmparc* atlas [8]. Semi-quantitative mean standardized uptake values (SUVs) were estimated for brain segmentations representing a range of anterior and posterior cerebral structures (anterior grey and white matter, posterior grey and white matter, deep grey matter, corpus callosum (whole), and the splenium of the corpus callosum). The structures assessed were as follows: anterior - the frontal lobe including pars opercularis/orbitalis/triangularis; posterior - occipital lobe including cuneus and lingual gyri; deep grey – thalamus, caudate, putamen, pallidum, accumbens area; corpus callosum – whole and splenium. White matter regions were defined by the nearest cortical region [8].

**References**

1. Karunanithi S, Soundararajan R, Sharma P, Naswa N, Bal C, Kumar R. Spectrum of Physiologic and Pathologic Skeletal Muscle (18)F-FDG Uptake on PET/CT. AJR Am J Roentgenol. 2015;205(2):W141-9. doi: 10.2214/AJR.14.13457. PubMed PMID: 26001118.

2. Kumar P, Patel CD, Singla S, Malhotra A. Effect of duration of fasting and diet on the myocardial uptake of F-18-2-fluoro-2-deoxyglucose (F-18 FDG) at rest. Indian J Nucl Med. 2014;29(3):140-5. Epub 2014/09/12. doi: 10.4103/0972-3919.136559. PubMed PMID: 25210278; PubMed Central PMCID: PMCPMC4157186.

3. van den Hoff J, Lougovski A, Schramm G, Maus J, Oehme L, Petr J, et al. Correction of scan time dependence of standard uptake values in oncological PET. EJNMMI Res. 2014;4(1):18. doi: 10.1186/2191-219X-4-18. PubMed PMID: 24693879; PubMed Central PMCID: PMCPMC3992152.

4. Keereman V, Fierens Y, Broux T, De Deene Y, Lonneux M, Vandenberghe S. MRI-based attenuation correction for PET/MRI using ultrashort echo time sequences. Journal of nuclear medicine : official publication, Society of Nuclear Medicine. 2010;51(5):812-8. doi: 10.2967/jnumed.109.065425. PubMed PMID: 20439508.

5. Laothamatas J, Sammet CL, Golay X, Van Cauteren M, Lekprasert V, Tangpukdee N, et al. Transient lesion in the splenium of the corpus callosum in acute uncomplicated falciparum malaria. Am J Trop Med Hyg. 2014;90(6):1117-23. Epub 2014/03/13. doi: 10.4269/ajtmh.13-0665. PubMed PMID: 24615139; PubMed Central PMCID: PMCPMC4047739.

6. Frolich AM, Tober-Lau P, Schonfeld M, Brehm TT, Kurth F, Vinnemeier CD, et al. Brain magnetic resonance imaging in imported malaria. Malar J. 2019;18(1):74. doi: 10.1186/s12936-019-2713-2. PubMed PMID: 30871543; PubMed Central PMCID: PMCPMC6419340.

7. Bourgeat P, Villemagne VL, Dore V, Brown B, Macaulay SL, Martins R, et al. Comparison of MR-less PiB SUVR quantification methods. Neurobiol Aging. 2015;36 Suppl 1:S159-66. doi: 10.1016/j.neurobiolaging.2014.04.033. PubMed PMID: 25257985.

8. Salat DH, Greve Dn Fau - Pacheco JL, Pacheco Jl Fau - Quinn BT, Quinn Bt Fau - Helmer KG, Helmer Kg Fau - Buckner RL, Buckner Rl Fau - Fischl B, et al. Regional white matter volume differences in nondemented aging and Alzheimer's disease. (1095-9572 (Electronic)).


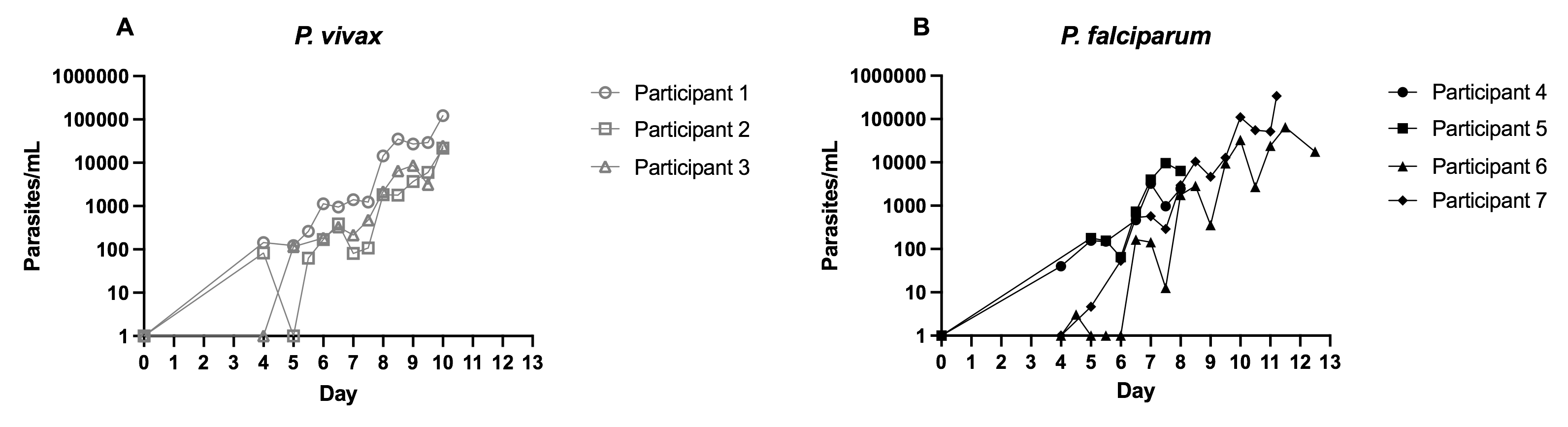


**Supplementary Figure S1:** Parasitemia curves for participants inoculated with A) *P. vivax* and B) *P. falciparum*

**Supplementary Table S1:** FDG dose and uptake time scans for study population. FDG: 18-F fluorodeoxyglucose, MBq/kg: megabecquerels per kilogram)

| **Challenge Agent** | **Participant** | **Baseline imaging** | | **Post-inoculation imaging** | |
| --- | --- | --- | --- | --- | --- |
|  |  | **FDG dose (MBq/kg)** | **Uptake time (minutes)** | **FDG dose (MBq/kg)** | **Uptake time (minutes)** |
| *P.vivax* | 1 | 4.2 | 136.8 | 4.2 | 122.9 |
|  | 2 | 4.6 | 136.7 | 4.4 | 129.4 |
|  | 3 | 4.3 | 123.5 | 4.6 | 157.8 |
| *P.falciparum* | 4 | 3.0 | 64.3 | 4.0 | 65.3 |
|  | 5 | 4.1 | 116.6 | 3.9 | 114.2 |
|  | 6 | 4.2 | 113.9 | 4.1 | 112.0 |
|  | 7 | 3.5 | 116.1 | 3.6 | 112.3 |

**Supplementary Table S2:** Quantitative neuroimaging metrics. SUV: mean standardized uptake value (no units), ADC: apparent diffusion coefficient (×10^-6^ mm^2^/s), BL: baseline, PI: post-inoculation, %Δ: percentage change from baseline

| **Participant** | **Challenge Agent** | **Anterior Grey Matter SUV** | | | **Anterior White Matter SUV** | | | **Posterior Grey Matter SUV** | | | **Posterior White Matter SUV** | | |
| --- | --- | --- | --- | --- | --- | --- | --- | --- | --- | --- | --- | --- | --- |
|  |  | **BL** | **PI** | **%Δ** | **BL** | **BL** | **%Δ** | **BL** | **PI** | **%Δ** | **BL** | **PI** | **%Δ** |
| 1 | *P. vivax* | 14.0 | 13.9 | -0.6 | 10.5 | 10.2 | -3.6 | 13.9 | 13.9 | +0.2 | 10.9 | 10.9 | +0.1 |
| 2 | *P. vivax* | 10.7 | 10.9 | +2.2 | 8.2 | 8.4 | +1.5 | 10.9 | 11.2 | +3.3 | 9.0 | 9.2 | +2.5 |
| 3 | *P. vivax* | 10.2 | 11.1 | +9.3 | 7.4 | 8.1 | +9.8 | 9.4 | 10.4 | +11.6 | 7.5 | 8.3 | +11.3 |
| 4 | *P. falciparum* | 9.0 | 12.2 | +35.2 | 6.8 | 9.5 | +38.9 | 8.8 | 12.2 | +38.8 | 7.1 | 9.8 | +38.7 |
| 5 | *P. falciparum* | 11.3 | 13.5 | +18.6 | 8.6 | 10.1 | +18.2 | 11.9 | 13.8 | +16.0 | 9.8 | 11.2 | +14.8 |
| 6 | *P. falciparum* | 8.0 | 7.9 | -0.7 | 6.4 | 6.6 | +4.3 | 7.4 | 7.8 | +5.3 | 6.0 | 6.6 | +8.9 |
| 7 | *P. falciparum* | 12.1 | 9.5 | -21.6 | 10.2 | 7.6 | -25.6 | 11.2 | 9.1 | -18.3 | 9.4 | 7.7 | -19.0 |

| **Participant** | **Challenge Agent** | **Deep Grey Matter SUV** | | | **Corpus Callosum SUV** | | | **Splenium SUV** | | | **Splenium ADC** | | |
| --- | --- | --- | --- | --- | --- | --- | --- | --- | --- | --- | --- | --- | --- |
|  |  | **BL** | **PI** | **%Δ** | **BL** | **BL** | **%Δ** | **BL** | **PI** | **%Δ** | **BL** | **PI** | **%Δ** |
| 1 | *P. vivax* | 12.5 | 12.2 | -2.4 | 6.7 | 6.6 | -1.7 | 7.2 | 7.0 | -3.5 | 756 | 817 | +8.1 |
| 2 | *P. vivax* | 9.2 | 9.2 | +0.2 | 4.2 | 4.3 | +1.9 | 4.5 | 4.5 | -0.5 | 677 | 754 | +11.4 |
| 3 | *P. vivax* | 8.3 | 9.2 | +10.1 | 3.9 | 4.3 | +9.3 | 4.2 | 4.5 | +7.8 | 843 | 749 | -11.2 |
| 4 | *P. falciparum* | 8.0 | 10.6 | +31.9 | 3.7 | 5.2 | +40.5 | 3.6 | 5.0 | +38.0 | 786 | 795 | +1.1 |
| 5 | *P. falciparum* | 9.9 | 11.4 | +14.9 | 3.9 | 4.6 | +20.1 | 4.2 | 5.1 | +21.2 | 622 | 795 | +27.8 |
| 6 | *P. falciparum* | 7.5 | 7.7 | +1.9 | 3.6 | 3.9 | +9.0 | 3.6 | 4.0 | +9.4 | 728 | 705 | -3.2 |
| 7 | *P. falciparum* | 10.4 | 8.2 | -20.8 | 5.3 | 4.4 | -16.7 | 5.1 | 4.4 | -14.5 | 740 | 713 | -3.6 |

**Supplementary Tabl**e **S3:** Comparison of mean (95% confidence interval) percentage change from baseline in quantative neuroimaging metrics for *P. falciparum* and *P. vivax* groups. SUV: mean standardized uptake value, ADC: apparent diffusion coefficient.

|  | ***P. vivax*** | ***P. falciparum*** | **P value** |
| --- | --- | --- | --- |
| Anterior grey matter SUV | +3.6% (-9.1% to +16.4%) | +7.9% (-31.2% to +46.9%) | 0.785 |
| Anterior white matter SUV | +2.6% (-14.2% to +19.4%) | +9.0% (-34.1% to +52.0%) | 0.714 |
| Posterior grey matter SUV | +5.1% (-9.6% to +19.7%) | +10.4% (-27.3% to +48.2%) | 0.722 |
| Posterior white matter SUV | +4.6% (-9.9% to +19.2%) | +10.9% (-26.9% to +48.6%) | 0.682 |
| Deep grey matter SUV | +2.6% (-13.8% to +19.0%) | +7.0% (-28.4% to +42.3%) | 0.759 |
| Corpus callosum SUV | +3.1% (-10.8% to +17.1%) | +13.2% (-24.7% to +51.1%) | 0.515 |
| Splenium SUV | +1.2% (-13.3% to +15.9%) | +13.5% (-21.6% to +48.6%) | 0.400 |
| Splenium ADC | +2.8% (-27.5% to +33.0%) | +5.5% (-18.3% to +29.4%) | 0.805 |
